# A Flexible and Highly Sensitive Inductive Pressure Sensor Array Based on Ferrite Films

**DOI:** 10.3390/s19102406

**Published:** 2019-05-27

**Authors:** Xinran Tang, Yihui Miao, Xinjian Chen, Baoqing Nie

**Affiliations:** 1 School of Electronic and Information Engineering, Soochow University, Suzhou 215006, China; 20165228032@stu.suda.edu.cn (X.T.); 20175228011@stu.suda.edu.cn (Y.M.); 2 State Key Laboratory of Radiation Medicine and Protection, Soochow University, Suzhou 215123, China

**Keywords:** flexible electronics, inductive sensing, pressure sensor array, wearable device

## Abstract

There is a rapid growing demand for highly sensitive, easy adaptive and low-cost pressure sensing solutions in the fields of health monitoring, wearable electronics and home care. Here, we report a novel flexible inductive pressure sensor array with ultrahigh sensitivity and a simple construction, for large-area contact pressure measurements. In general, the device consists of three layers: a planar spiral inductor layer and ferrite film units attached on a polyethylene terephthalate (PET) membrane, which are separated by an array of elastic pillars. Importantly, by introducing the ferrite film with an excellent magnetic permeability, the effective permeability around the inductor is greatly influenced by the separation distance between the inductor and the ferrite film. As a result, the value of the inductance changes largely as the separation distance varies as an external load applies. Our device has achieved an ultrahigh sensitivity of 1.60 kPa^−1^ with a resolution of 13.61 Pa in the pressure range of 0–0.18 kPa, which is comparable to the current state-of-the-art flexible pressure sensors. More remarkably, our device shows an outstanding stability when exposed to environmental interferences, e.g., electrical noises from skin surfaces (within 0.08% variations) and a constant pressure load for more than 32 h (within 0.3% variations). In addition, the device exhibits a fast response time of 111 ms and a good repeatability under cyclic pressures varying from 38.45 to 177.82 Pa. To demonstrate its practical usage, we have successfully developed a 4 × 4 inductive pressure sensor array into a wearable keyboard for a smart electronic calendar application.

## 1. Introduction

Flexible sensing has attracted considerable attention in both academia and industries with a wide range of applications in smart wearable devices for human–machine interactions [1,2,3,4,5]. Various sensing mechanisms are exploited, including resistance, capacitance, piezoelectricity, optics and triboelectricity [6,7,8,9,10,11]. Among those, inductive sensors are widely used in the fields of displacement measurements, particle detections, and health monitoring because of their high resolution, long life time, good linearity, high stability, simple structure and immunity to environmental fluctuations [12,13,14,15,16]. According to the law of electromagnetic induction, an inductor can generate a magnetic field as current flows through it. The parameters, such as self-inductance coefficient, relative permeability and magnetic flux distribution, can be modified by external stimuli [14,15,16]. For instance, Kisic’s group has reported a method to detect seat occupancy by using an inductive-based wireless pressure sensor. Body weight applied to the sensor causes the compressions of built-in springs, inducing the decrease in the distance between an inductor and a ferrite plate, which in turn changes in the sensor’s inductance, consequently, the resonant frequency of the antenna changes [14]; Prof. Zhe’s group has reported an inductive sensor for detecting micro-scaled metallic debris in lubrication oil. A microfluidic channel was inserted in the center of two parallel planar coils, and the overall inductance of the coils varies as the fluid with metallic particles in the channel passed through [15]. Recently, with the rapid development of new functional materials, inductive sensors get improved in many aspects, including the fabrication process, structural configuration and device performance. For instant, Prof. Lee’s group has reported an inductor-capacitor (LC)-based wireless pressure sensor, in which the inductance is determined by the pressure-dependent distance between a micro-coil and upper materials (e.g., ferrite or metal). The device has proven a robust output signal in in vitro demonstration for intraocular pressure measurement. [16].

Recently, there is an emerging attention in broad applications of pressure sensing devices/systems, ranging from health monitoring, human physiological signal detection, artificial skin, to prosthetic surgeries [17,18,19,20]. In many cases, pressure sensors convert the physical forces/pressures to structural deformations, which further lead to the variations of electrical signals. For example, Prof. Zhou’s group has reported a self-powered piezoelectric sensor by translating the pressure into the variations in the electrical potential of an outer sheath and an inner core based on the electrostatic effect, achieving a high device sensitivity of 18.98 V·kPa^−1^ in the pressure range of 0–0.5 kPa [21]. Functional materials, such as graphene, graphite and their oxides, with excellent electrical conductivity and robust mechanical reliability, are widely used in flexible pressure sensors. Prof. Dong’s group has reported a flexible piezoresistive pressure sensor by filling sponge with reduced graphene oxide (rGO) and polyaniline nanowires (PANI NWs), in which the rGO ensures the excellent conductivity while the PANI NW increases the contact areas in the sponge. The sensor has a pressure sensing range of 0–3.24 kPa with a sensitivity of 0.152 kPa^−1^. It has been used to detect tiny human motions (e.g., voice recognition, breath) and large-scale body activities (e.g., finger bending, elbow and knee movements) [22]. Nano-scaled structures are introduced into sensors to increase the surface roughness and therefore improve the device sensitivity. In a recent report, Prof. Shim and co-workers proposed a high-transparency capacitive pressure sensor for artificial blood vessels, flexible keyboard, etc. The sensor is built on a polydimethylsiloxane (PDMS)-SiO_2_ nanoparticle composite sandwiched by two electrode layers. SiO_2_ nanoparticles enhance the surface roughness of PDMS to increase the compressibility and capacitive sensitivity. The sensor has high sensitivity of 1.0 kPa^−1^ within 2 kPa [23]. The triboelectric pressure sensor is one of the most effective devices for converting ambient mechanical energy to electrical energy. Prof. Ko’s group has reported a wearable self-powered triboelectric pressure sensor by stitching polyvinylidene fluoride (PVDF) fibers into patterns. The sensor exhibits sensitivity of 0.66 nA·kPa^−1^ in the pressure range of 0–16.3 kPa and it has excellent washability. The device can be widely applied to hand gesture detection and real-time pulse monitoring [24].

**Figure 1:**
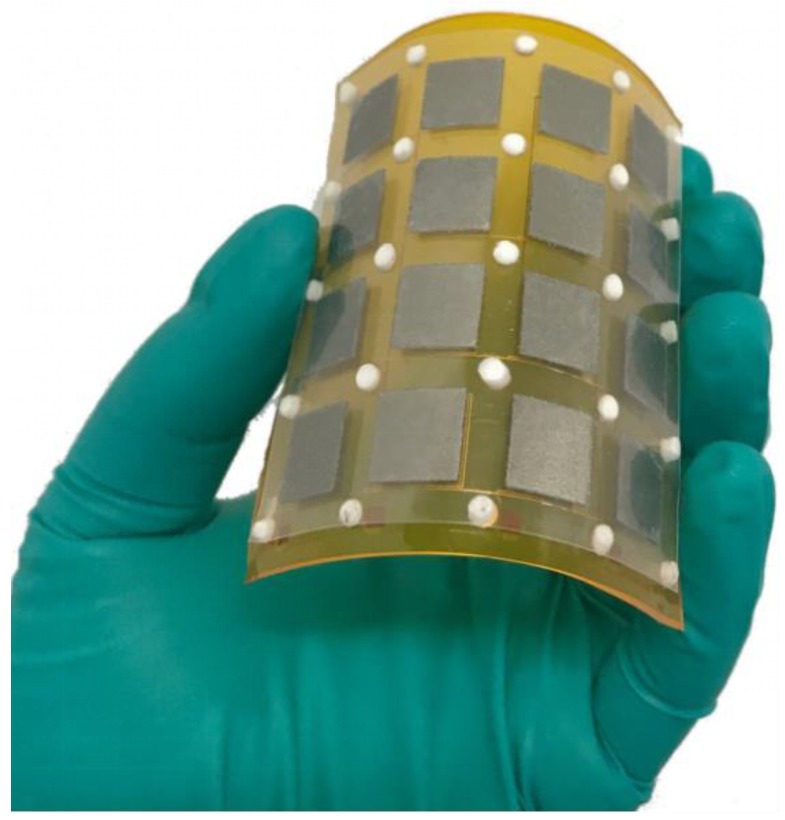
A photograph of a 4 × 4 inductive pressure sensor array.

In this paper, we report an innovative flexible inductive pressure sensor array by introducing a unique ferrite material with an excellent permeability for emerging wearable sensing applications. Figure 1 illustrates a 4 × 4 flexible inductive pressure sensor array, of which a piece of ferrite film implemented on a simply suspended polyethylene terephthalate (PET) membrane structure in each sensing unit, effectively enhancing the inductance value of the planar inductor. The flexible deformation of the ferrite film allows the variation in the distance between the film and the planar inductor in response to mechanical stimuli, which in return changes the overall unit inductance. The inductive pressure sensor array achieves a high device sensitivity of 1.60 kPa^−1^ along with an ultrahigh resolution of 13.61 Pa, which is higher than most of inductive pressure sensors reported previously. Importantly, to optimize the device performance, key design parameters, i.e., the side length of the ferrite film/copper coils and the overall thickness of the ferrite film/PET membrane, were thoroughly characterized by theoretical analyses and experimental investigations. Additionally, the response time (of 111 ms) was characterized under a constant external load of 107 Pa. The device also exhibits an outstanding long-term stability to a constant pressure at different exciting voltages (within 0.3% in the inductance changes), rendering our sensor very competitive compared with the polymer-based piezoresistive pressure/stress sensors. Our sensor also shows great immunity to environmental interferences (i.e., human bioelectricity or electromagnetic interference). To demonstrate the potential utility in flexible electronics for our simply constructed and flexible inductive pressure sensor array, we successfully developed the sensor into flexible electronic gadgets, i.e., a wearable smart calendar touch board/keyboard. We believe the simple-constructed and ultra-high sensitive pressure sensing array offers an alternative solution in wearable electronics.

The contents are arranged as follows: in Section 2, we address the device fabrication process and experimental setup for the performance characterization. The results and discussions are presented on Section 3, including the working principle, sensitivity, minimum detectable pressure, repeatability, response/recovery time, stability and the demonstration for potential application in wearable electronics.

## 2. Materials and Methods

Sensor Fabrication: The general process of device fabrication includes three steps: planar inductor fabrication, laser micromachining and device assembly. In the first step, we patterned a double-sided copper-clad polyimide (PI) film using the standard screen-printing method and a wet etching process. Additional alignment markers on the PI film were created in this step. These markers were used to locate the positions of elastic pillars in the following steps. In the second step, direct laser micromachining was employed to cut the ferrite films (100 μm in thickness, A4010, Nanjing Advanced Magnetic Material Co., Ltd., Nanjing, China) and polyethylene terephthalate (PET) substrate (50 and 125 μm in thickness) with alignment markers. Elastic cylindrical pillars (2 mm in thickness) were cut on double-sided adhesive tape (Shenzhen Changda Sheng Electronics Co., Ltd., Shenzhen, China) by a punch with diameter of 3 mm. In the final assembly, elastic cylindrical pillars were aligned to the alignment markers on the PI film, and the ferrite film was attached on the PET substrate, followed by assembling the ferrite/PET film to the adhesive pillars. Electrical wires were connected to the contact pads for measurements of the inductance values. The whole device was soft and flexible, with customized dimensional size of 17.6 × 17.6 × 2.2 mm (smallest) to 27.0 × 27.0 × 2.2 mm (largest) of each sensing unit.

**Figure 2:**
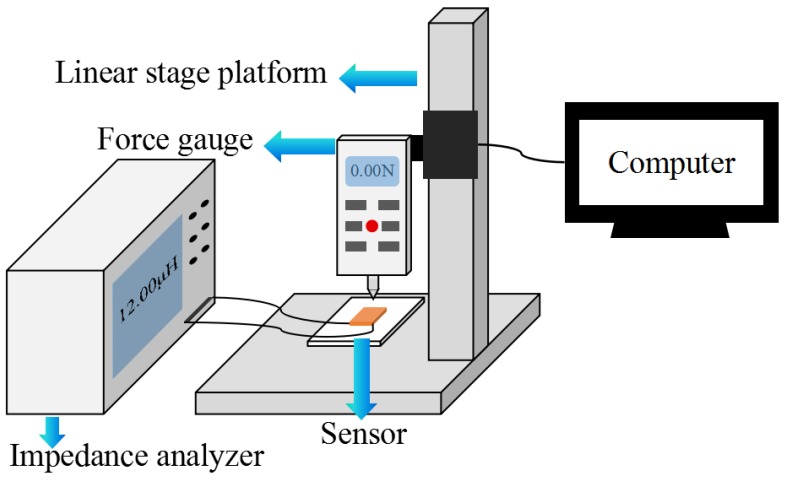
The measurement setup for the device calibrations.

Device Characterization: For the sensitivity calibration, external mechanical point loads were applied onto the center of the ferrite/PET film through a custom-built motorized force gauge with 1 mN resolution (M5-05, Mark10, Inc., Copiague, NY, USA), driven by a computer-controlled linear stage platform (LTS300/M, Thorlabs Inc., Newton, NJ, USA) with a spatial resolution of 0.1 µm. The pressure was determined by the ratio of the force and the area of ferrite film/copper coil in each sensing unit. The inductance of the device was assessed electrically by using an impedance analyzer (65120B, Wayne Kerr Electronics Co., Ltd., London, UK), in which an AC excitation voltage of 1 V and 1 kHz was applied to the device and the inductance was recorded. For the resolution measurement, minute pressure was applied onto the ferrite/PET film via tiny displacement of the linear stage platform till a noticeable inductive change appeared in the LCR meter. The schematic diagram of the characterization system is shown in Figure 2.

## 3. Results and Discussion

### 3.1. Sensitivity and Minimum Detectable Pressure

**Figure 3:**
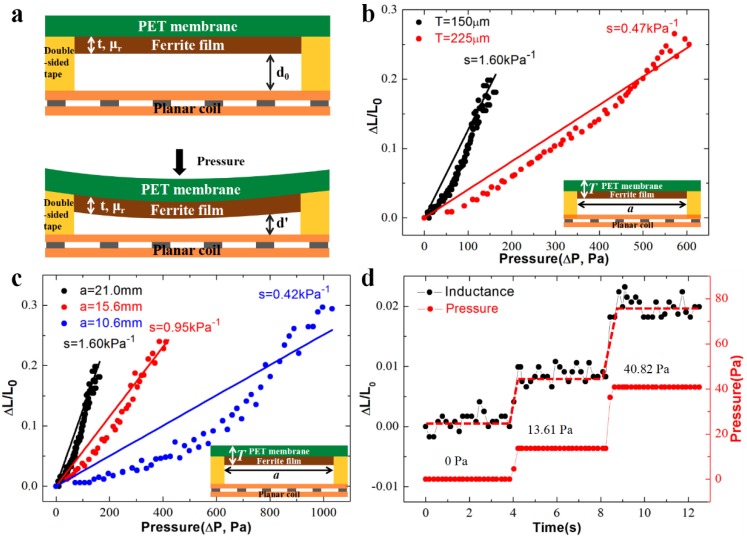
(**a**) Schematic diagram of operation principle of the inductance pressure sensor in a cross-sectional view; (**b**) experimental and theoretical investigations on the device sensitivity as the thickness of the polyethylene terephthalate (PET)/ferrite film *T* varying from 150 to 225 μm and (**c**) the edge length of the ferrite film *a* changing from 10.6, 15.6 to 21.0 mm. The measurement results (dots) are plotted against the theoretical predictions (solid lines) from Equation (2); (**d**) the inductance responses to an incremental pressure from 0 to 40.8 Pa.

Figure 3a illustrates the cross-sectional view of one sensing unit of the inductive pressure sensor array, which consists of the top layer of ferrite film attached on PET membrane and the bottom planar coil, separated by elastic pillars. Owning to the ultra-high permeability (*μ_r_*) of the ferrite film, the inductance of the planar coil is greatly enhanced due to the increase in the effectively permeability of the surrounding environment. The effective inductance (*L*_0_) follows the formula [25]:

(1) $$L_{0}=\frac{N^{2}\mu_{0}}{\frac{l_{f}}{A_{f}\mu_{r}}+\frac{l_{a}}{A_{a}}},$$

where *N* is the number of turns of the copper coils, *μ*_0_ is the vacuum permeability. *l_f_* and *A_f_* are the length and the area of the magnetic circuit dissipated in the ferrite film, which are largely determined by the geometrical dimension of the ferrite film. *l_a_* and *A_a_* represent the length and the area of the magnetic path in the air, which is related to the distance between ferrite film and planar spiral inductor (*d*_0_) [26].

As an external pressure applies to the sensor membrane, the deformation of the suspended ferrite/PET film leads to the decrease in the separation distance from *d*_0_ to *d*’, which further increases on the overall inductance. The relationship of the relative inductive change (Δ*L*/*L*_0_) and the pressure (Δ*P*) is expressed as [27,28]:

(2) $$\frac{\Delta L/L_{0}}{\Delta P}=\frac{a^{2}\left(1-\nu^{2}\right)}{5ET^{3}\left(\frac{1}{t\mu_{r}+C}\right)},$$

where *a* and t are the side length of the ferrite film/copper coils and the thickness of the ferrite film, respectively; *v*, *E* and *T* are the effective Poisson’s ratio, Young’s modulus and the total thickness of the ferrite/PET membrane, respectively; *C* is a constant which represents an approximate magnetic pass length in the air excluded the space between the inductor and ferrite film (see Supplementary Materials for more details). According to Equation (2), the higher the permeability of the ferrite film (*μ_r_*) is, the larger the inductance changes under a constant pressure, indicating the device sensitivity can be improved by using a ferrite film with a higher *μ_r_*. In addition, the total thickness of the PET/ferrite membrane (*T*) and the side length of the ferrite film (*a*) are two important factors which have great influences on the device sensitivity, considering that the value of Δ*L*/*L*_0_/Δ*P* is inversely proportional to the third power of the *T* and linear to the second power of the *a*.

Both the theoretical analysis and experimental validations are performed to investigate the device sensitivity. According to Equation (2), the geometrical parameters, such as the total thickness of ferrite/PET film (*T*) and the edge length of the copper coils (*a*), the young modulus (*E*), and relative permeability of the ferrite film (*μ_r_*) influence the overall sensitivity. Among those, the sensitivity is most influenced by the total ferrite/PET film thickness (*T*, by 3rd power), followed by the 2nd power of the edge length (*a*). Here we choose the two key parameters as the variable design factors to investigate the impacts of the design parameters on the device sensitivity.

Figure 3b illustrates the relative inductance change (Δ*L*/*L*_0_) over loaded pressures (Δ*p*) on one sensing unit with two different ferrite/PET film thicknesses, i.e., 150 and 225 μm, provided a constant edge length of ferrite film/copper coils of 21.0 mm. The experimental measurements (dots) are plotted in comparison with the values calculated from Equation (2) (the solid lines show the results of theoretical prediction), and the slope rate of each device defines the corresponding device sensitivity (*s* = Δ*L*/*L*_0_/Δ*p*). As shown, the thinner ferrite/PET film (150 μm) shows a higher sensitivity (1.60 kPa^−1^), in comparison with the sensitivity of 0.47 kPa^−1^ in the thicker film (225 μm) design. The result closely follows the negative cubic relationship between the sensitivity and the ferrite/PET film thickness. In addition, the influence of edge length of the ferrite film/copper coils, varying from 10.6 to 21.0 mm on the device sensitivity was also investigated (Figure 3c). All the devices have a constant film thickness of 150 μm. In the devices with the largest edge length (of 21.0 mm), the highest sensitivity of 1.60 kPa^−1^ is achieved. In comparison, as the length is reduced to 10.6 mm, the system sensitivity drops drastically to less than 0.42 kPa^−1^. Those data suggest that the theoretical model fits the experiments reasonably well: the edge length influences the sensitivity by 2nd power. However, there is a compromise between the device sensitivity and the linear dynamic range: although the device sensitivity decreases as the ferrite/PET film thickness increases or the edge length decreases, the linear pressure range is further improved. For example, the sensor with a ferrite/PET membrane thickness of 150 μm and the ferrite film edge length of 10.6 mm shows a lowest sensitivity of 0.42 kPa^−1^, but it has the largest linear pressure range of 0–1 kPa. In addition, the sensitivity and the dynamic range of the pressure sensor in different bending states were also investigated (Figure S1 in Supplementary Materials). Different bending radii of curvatures post minimal influence on the device sensitivity, whereas the linear dynamic range becomes narrow as the bending radius of the curvature decreases. Moreover, the minimum detectable pressure was determined. As shown in Figure 3d, our sensor with 150 μm in the thickness of the PET/ferrite film and 21.0 mm in the edge length of the ferrite film has an inductance change of 0.89% as the external pressure increases from 0 to 13.61 Pa, and the changes reach to 1.15% as the pressure increases from 13.61 to 40.82 Pa. The result indicates that our senor is able to detect the pressure as low as 13.61 Pa, which is better than most reported inductive pressure sensors to the best of our knowledge.

### 3.2. Repeatability, Response/Recovery Time and Stability

**Figure 4:**
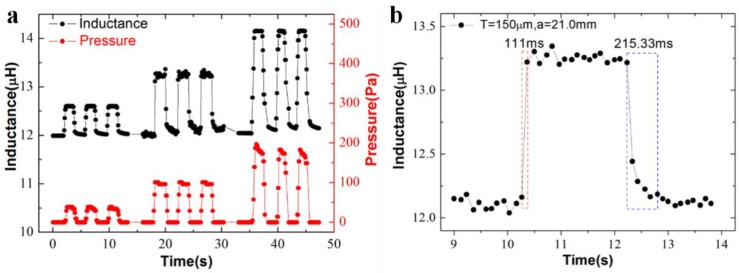
Characterization of the repeatability on the device with 150 μm in the thickness of the PET/ferrite film and 21.0 mm in the edge length of the ferrite film. (**a**) Inductive changes as a function of repetitive cycles of external pressures varying from 38.45, 107.00 to 177.82 Pa; (**b**) time-resolved inductive responses to repetitive mechanical loads of 107 Pa, from which the response time (111 ms) and the recovery time (215.33 ms) are evaluated from the rising and falling edges.

The device repeatability was evaluated under three different periodically mechanical loads (38.45, 107.00 and 177.82 Pa) as illustrated in Figure 4a. For all the repeated cycles in different pressure ranges, the sensor is able to sensitively respond to the cyclic loads and return to its original inductive value. The results indicate a reliable reproducibility of our sensor within the applied force ranges.

The response/recovery time was determined by analysis of the sensor outputs under press and release cycles. Figure 4b exhibits the time-resolved sensor responses to a load of 107 Pa in one cycle, in which the times spent on the rising and falling edges of the response curve are 111 and 215.33 ms respectively, from which the response/recovery time can be estimated. In addition, the repeatability and response/recovery time on another sensor design, i.e., the one with the lowest sensitivity (*T* = 150 μm and *a* = 10.6 mm), was also investigated (Figure S2 in Supplementary Materials). The results suggest that the design parameters *T* and *a* have little influence on the repeatability and response/recovery time.

**Figure 5:**
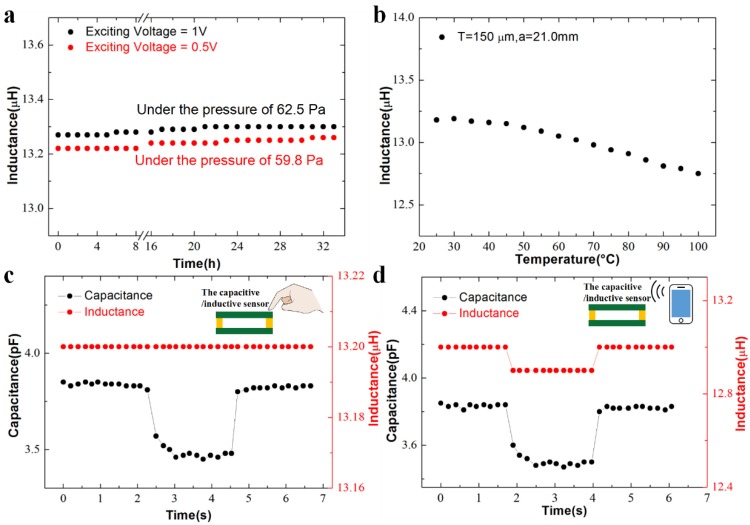
Characterization of the device stability. The output responses of the sensor with a film thickness of 150 μm and an edge length of 21.0 mm to (**a**) a constant pressure over 32 h at different exciting voltages of 1 and 0.5 V. (**b**) Temperature varying from 25 to 100 °C, (**c**) the human finger in comparison with the performance of a capacitive pressure sensor with a same dimension, and (**d**) an electromagnetic interference (EMI) device in comparison with the performance of the same capacitive pressure sensor. The exciting voltage in experiments of (**b**–**d**) is 1 V.

Time-resolved experiments were performed for device stability by applying a constant load, and the sensor output was recorded for more than 32 h. The inductance outputs were acquired every 30 min. As shown in Figure 5a, our sensor remains stable values at two different exciting voltages of 1 and 0.5 V, in which the maximum variations of the inductance over 32 h are 0.23% and 0.30%, respectively, which suggests an excellent long-term stability in practical usages, especially compared with the piezeresistive-based stress/pressure sensors [29,30,31].

In a practical wearable application, the device could be exposed to human body or even attached to human skin. The potential influences of human skin, in terms of temperature or electrical potential, on the device performance should be considered. Here, we recorded the inductive changes as the temperature varies from room temperature (25 °C) to an elevated level (100 °C). The result is shown in Figure 5b. The output of the inductive sensor decreases about 3.41% as the temperature increases to 100 °C. However, as the device is worn on human body, where the temperature usually ranges up to 40 °C, there is no significant change in the output (0.23%) under this condition. In addition, we recorded time-resolved inductive changes as fingers approached the sensor. In contrast, we also recorded the capacitance outputs of a capacitive pressure sensor with a similar structure: a parallel-plate capacitor constructed on two flexible electrodes separated with four elastic pillars. As shown in Figure 5c, our inductive pressure sensor keeps relatively stable output with a negligible variation of 0.076%, suggesting an excellent stability to the surrounding interference. The sensor with another design parameter was also proven to have an excellent interference-free ability to the presence of fingers (Figure S3 in Supplementary Materials). However, the output of the capacitive sensor decreases about 9.87% as the finger approaches to the sensor.

In addition, we also investigated electromagnetic interference (EMI) on the inductive and capacitive sensors. Figure 5d illustrates the effects of EMI from a mobile phone on the two devices. The output of our inductive pressure sensor decreases about 1.07%, whereas the output of the capacitive sensor decreases about 9.09% as the mobile phone approaches the sensors. The result indicates that EMI devices have less influence on the inductive sensor, which promises wide wearable applications for human-motion monitoring.

Table 1 compares the performances of our ferrite film-based inductive pressure sensor to current state-of-the-art flexible pressure sensing devices. Diversified research efforts have been attempted in the developments of soft pressure detections, most of which rely on resistive, capacitive, piezoelectric or inductive sensing mechanisms. Appealing performances have been achieved, including high sensitivity, low detectable pressure, fast response time and large linear range. Among those, our inductive sensor is very competitive in the device sensitivity and minimum detectable pressure. The sensing range is narrow than others, which could be improved by increasing the thickness of the separating pillars. The response time of our device is longer than some excellent designs in the table, which highly attributes to the viscoelastic properties of the separation pillars. However, our device shows an outstanding stability under long-time loads and different temperature conditions. Our device is also attractive considering its great immunity to the interferences of electrical potential from human body or electromagnetic field from mobile phones. The improvements in the future may include the materials with a fast mechanical response or new operating designs for better performance in the device.

### 3.3. Smart Wearable Keyboard

**Figure 6:**
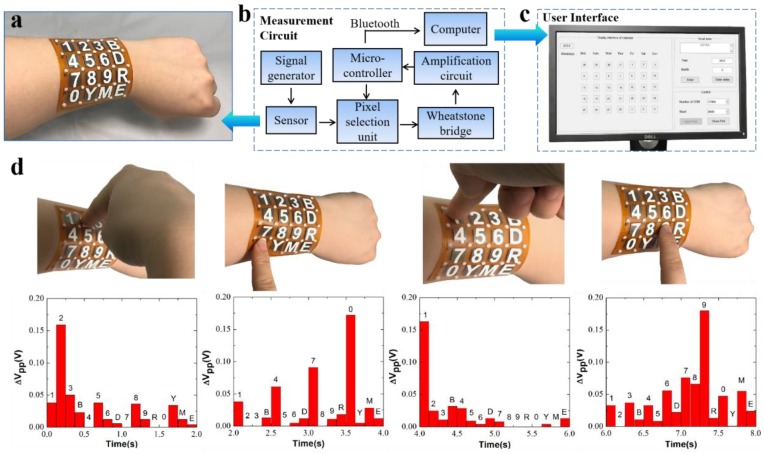
Demonstration of the utility of the flexible inductive pressure sensor array. (**a**) A wearable sensor prototype is attached on the forearm, in which each unit corresponds to a unique character. (**b**) The schematic illustration of the measurement circuit system. (**c**) A programmable user-interface display in a PC. (**d**) The voltage changes when four units of ‘2’, ‘0’, ‘1’ and ‘9’ are pressed by the fingertip.

Our inductive pressure sensing scheme exhibits characteristics of high sensitivity, fast response time, excellent stability and repeatability, showing a great potential in the application in wearable electric gadgets. Here, we successfully demonstrated a smart wearable keyboard by using our flexible 4 × 4 inductive pressure sensor array. The sensor array with the surface area of 6.67 × 6.60 cm^2^ and thickness of 2.2 mm is attached to human body, e.g., the forearm. Each sensing unit with a membrane thickness of 150 μm and a sensing area of 10.6 mm represents a unique button. As illustrated in Figure 6a, the 16 buttons include the numbers 0 to 9, year (Y), month (M), backspace (B), delete (D) and enter (E).

To acquire the electrical outputs from the sensor array, a customized measurement circuit is built and the corresponding block diagram is illustrated in Figure 6b. In general, the circuit consists of a signal generation unit, a pixel selection unit, a Wheatstone bridge unit, a signal amplification unit and a data acquisition unit. All the individual pixels are addressed by two orthogonally controlled multiplexers, which are regulated by a microcontroller. The output voltage from the selected unit flows into the Wheatstone bridge, then into the signal amplification. The output voltages are acquired by the microcontroller and sent by Bluetooth to a computer (Figure 6c). The details on the measurement circuit are provided in Supplementary Materials.

Figure 6d illustrates the voltage changes of the 16 units (bottom figures) as the buttons of “2”, “0”, “1” and “9” are pressed (top figures). The typical pressure range we applied on the sensor was from 0.35 to 0.70 kPa. As shown, the corresponding units have largest voltage changes (more than 0.15 V) as they are pressed, which can be easily recognized in the following data process. It is noted that there is a signal cross-talking in adjacent units, which is highly likely to be due to the mechanical interferences from the PET membrane between units. To address this issue, materials of the sensing membrane and the elastic pillars with proper mechanical modulus should be investigated in future work.

We have further demonstrated our sensor in a smart calendar application. The voltage changes of the active units were recognized in the data processing and analysis. As a result, the input year of “2019” and month of “2” are captured and transferred to PC, and the corresponding electrical calendar is calculated and displayed on the PC in a MATLAB program.

## 4. Conclusions

In summary, this paper reports a highly sensitive flexible pressure sensor array by utilizing planar spiral inductors and ferrite films. The ferrite film with an ultra-high permeability effectively increases the inductance of the planar inductor as the separation distance decreases. Our device has achieved an ultrahigh sensitivity of 1.60 kPa^−1^ in the pressure range of 0–0.18 kPa. In addition, we demonstrated the sensor to have a fast response time (of 111 ms), an excellent minimal detection limit (of 13.61 Pa) and outstanding long-term stability (within 0.3% variation under a constant pressure over more than 32 h). As a conceptual proof for wearable sensing, we have successfully developed an electronic perpetual calendar by attaching our inductive pressure sensor array on the forearm. The result proves that it shows great potential in flexible electronic applications.

## Figures and Tables

**Table 1:** The comparisons of the device performances on the current state-of-art flexible pressure sensing devices.

Reference	Sensing Mechanism	Sensing Area (mm^2^)	Sensitivity (kPa^−1^)	Linear Range (kPa)	Minimum Detectable Pressure (Pa)	Response Time (ms)
[2]	Resistive	20 × 20	0.3	0–0.7	<20	~162
[32]	Piezoresistive	4 × 4	4.5 × 10^−2^	0–10	~15	~700
[33]	Piezoresistive	3 × 3	1.2	0–25	5	-
[34]	Piezoresistive	28 × 28	0.011	1–120	~1000	180
[35]	Piezoresistive	10 × 10	4.1	0–10	-	55
[36]	Piezoresistive	11 × 11	2.2	0.035–2.5	<35	35–40
[37]	Capacitive	4 × 4	3.8	0.05–0.5	15	<150
[38]	Capacitive	10 × 10	0.8	0–1	~0.24	~100
[39]	Capacitive	18 × 25	2.94	0–2	<3	<50
[40]	Inductive	1.78 × 1.78	7.9 × 10^−4^	0–300	1760	-
This work	Inductive	27.0 × 27.0	1.6	0–0.18	13.61	111

## Supplementary Materials

The following are available online at https://www.mdpi.com/1424-8220/19/10/2406/s1, Fugure S1: The relative inductance changes to the pressure under three different bending radii of curvatures, i.e., infinity, 48.2 and 35 mm, Figure S2: Characterization of the device repeatability, Figure S3: The sensor outputs responses as a finger moved toward the sensor surface, in comparison with the performances of a capacitive, Figure S4: Schematic diagram of the inductance measurement module, Figure S5: Schematic diagram of the amplification circuit unit, Equations (S1)–(S12): theoretical analyses on the device sensitivity.
