# Supplementary material for: A Flexible and Highly Sensitive Inductive Pressure Sensor Array Based on Ferrite Films
Source: Sensors (Basel). 2019 May 27;19(10):2406. doi: 10.3390/s19102406 (PMC6566628; doi:10.3390/s19102406)
Supplement: Supplementary file 1 [file sensors-19-02406-s001.pdf]

Supplementary Materials

A Flexible and Highly Sensitive Inductive Pressure Sensor Array Based on Ferrite Films

Xinran Tang [1], Yihui Miao [1], Xinjian Chen [1,2,*] and Baoqing Nie [1,*]

1 School of Electronic and Information Engineering, Soochow University, Suzhou 215006, China; 20165228032@stu.suda.edu.cn (X.T.); 20175228011@stu.suda.edu.cn (Y.M.)

2 State Key Laboratory of Radiation Medicine and Protection, Soochow University, Suzhou 215123, China

* Correspondence: xjchen@suda.edu.cn (X.C.); qixinbq@suda.edu.cn (B.N.)

Characterization of bending effect

We experimentally calibrated the device responses to the external pressures when it is bent under the radii of infinite, 48.2 and 35 mm. As shown in Figure S1, the results suggest that the device sensitivity changes marginally under different bending states. The dynamic sensing range decreases as the bending radius of the curvature reduces. It is likely that an initial mechanical deformation/compression of the spacer layer is induced as the device is bent, leading to less room for the sensing membrane to be pressed.

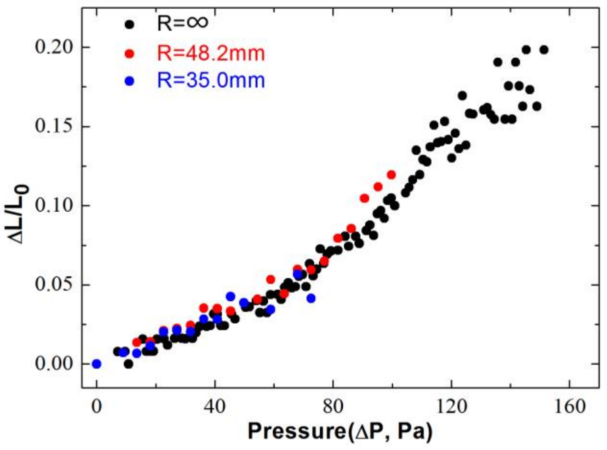

Figure S1. The relative inductance changes to the pressure under three different bending radii of curvatures, i.e., infinity, 48.2 and 35 mm.

Characterizations of Repeatability, Response/Recovery Time and Stability

The device repeatability and response/recovery times were evaluated on the device with the thickness of PET/ferrite film T of 150 μm and the edge length of the ferrite film a of 10.6 mm. The response/recover times are close to that of the sensor with T = 150 μm and a = 10.6 mm, suggesting that the design parameters T and a have little influence on the repeatability and response/recovery time.

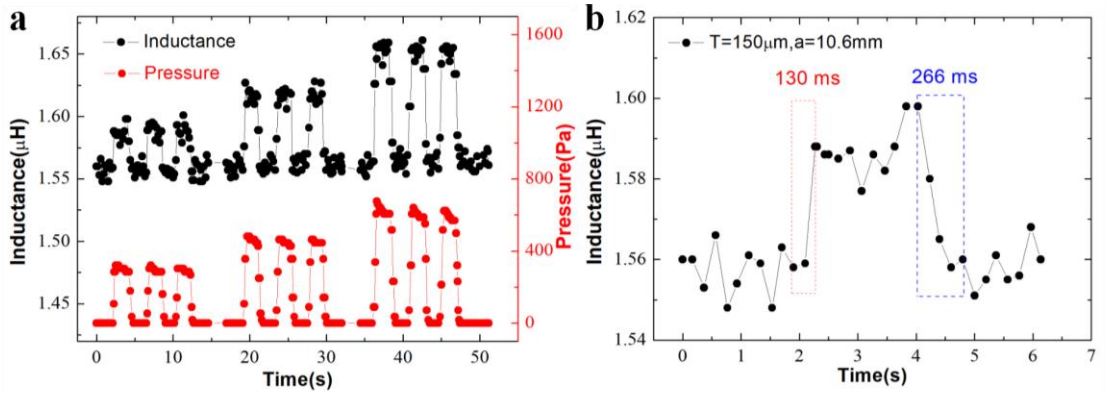

Figure S2. Characterization of the device repeatability. The thickness of the PET/ferrite film *T* is 150 μm and the edge length of the ferrite film *a* is 10.6 mm. (**a**) Inductive changes as a function of repetitive cycles of external pressures varying from 298.7, 476.7 to 645.3 Pa; (**b**) time-resolved inductive responses to repetitive mechanical loads of 298.7 Pa, from which the response time (130 ms) and the recovery time (266 ms) are evaluated from the rising and falling edges.

We also recorded time-resolved inductive changes as fingers approached the sensor with the same dimension. In addition, the capacitance outputs of a capacitive pressure sensor were also compared. As shown in Figure S3, the output variation of our inductive pressure sensor is 8.52%, and the variation for the capacitive sensor is 0.61%, suggesting an excellent stability of our sensor to the interference.

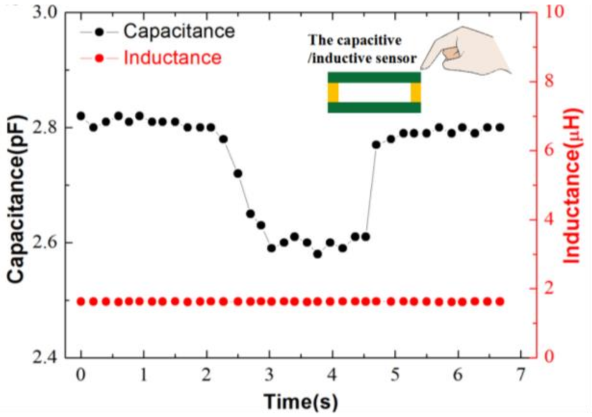

Figure S3. The sensor outputs responses as a finger moved toward the sensor surface, in comparison with the performances of a capacitive pressure sensor with the same dimensions. The thickness of the PET/ferrite film *T* and the edge length of the ferrite film *a* of the inductive sensor is 150 μm and 10.6 mm, respectively.

The Measurement Circuit for the Pressure Sensor Array

Our measurement circuit consists of a signal generation unit, a pixel selection unit, a Wheatstone bridge unit, a signal amplification unit and a data acquisition unit. The copper electrodes naturally have parasitic resistance. When an AC signal passes through the planar inductor, the parasitic resistance shares most of the input voltage, which brings difficulty in detecting the small variation in the inductance value. In order to detect the small inductive change, we used a Wheatstone bridge to balance the parasitic resistance of the electrode. Figure S4 shows the circuit diagram of the Wheatstone bridge.

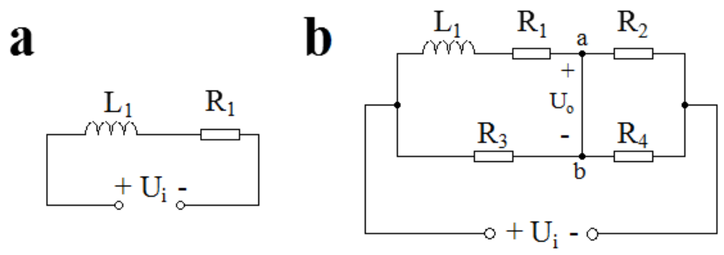

Figure S4. Schematic diagram of the inductance measurement module. (**a**) Equivalent electric diagram of a single sensing unit of the sensor array. (**b**) Circuit diagram of the Wheatstone bridge.

L_1 and *R_1* are the inductance and parasitic resistance of the sensor, respectively. *R_2*, *R_3* and *R_4* are fixed value resistors. Here, we take *R_1* = *R_3*, *R_2* = *R_4*. Therefore, the output voltages *U_o* can be obtained in Equation S1.

$$
\begin{aligned}
U_o = U_a - U_b &= \left(\frac{R_2}{Z_1 + R_1 + R_2} - \frac{R_4}{R_3 + R_4} \right) U_i \\
&= -\frac{Z_1 R_4}{(R_3 + R_4) Z_1 + R_1 R_3 + R_1 R_4 + R_2 R_3 + R_2 R_4} U_i
\end{aligned} \tag{S1}
$$

Following the Wheatstone bridge unit, we added a secondary amplifier circuit to amplify the output voltage by 1000 times. The schematic diagram is shown in Figure S5. The output voltages *U_{o1}* can be calculated in Equation S2.

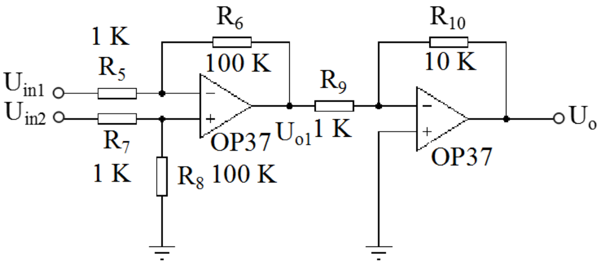

Figure S5. Schematic diagram of the amplification circuit unit.

$$
\begin{aligned}
U_{o1} &= \frac{R_5 + R_6}{R_7 + R_8} \cdot \frac{R_8}{R_5} \cdot U_{in2} - \frac{R_6}{R_5} \cdot U_{in1} \\
&= \left(U_{in2} - U_{in1} \right) \frac{R_6}{R_5}
\end{aligned} \tag{S2}
$$

After the signal entering the second amplifier circuit, the output voltage is:

$$\frac{U_o}{U_{in1} - U_{in2}} = \frac{R_{10}}{R_9} \cdot \frac{R_6}{R_5} \tag{S3}$$

The amplified AC signal is covered into a DC signal by a peak detection circuit. The DC signal is then sent to PC by the Bluetooth module after processed by the microcontroller.

Mathematical Derivations for the Mechanical-to-Inductive Sensitivity

Deformation-Induced Inductive Change

According to the basic knowledge of the magnetic circuit, the inductance value of the planar spiral inductor can be calculated by the formula [1]:

$$L = \frac{N^2}{R_m} \tag{S4}$$

,

where N is the turns of the coil and R_m is the equivalent reluctance. We assume that the magnetic field is mainly distributed in the ferrite film and the air gap, and the corresponding equivalent magnetic reluctances are R_f and R_a, respectively. The formula of R_m in Equation (S4) can be approximated as:

$$R_m = R_f + R_a = \frac{l_f}{\mu_0 \mu_r A_f} + \frac{l_a}{\mu_0 A_a} \tag{S5}$$

,

where l_f and A_f are the length and area of the magnetic circuit dissipated in the ferrite film, A_a and l_a represent the area and length of the magnetic path in the air [2]. Combining Equations (S4) and (S5), we have:

$$L_0 = \frac{N^2 \mu_0}{\frac{l_f}{A_f \mu_r} + \frac{l_a}{A_a}} \tag{S6}$$

.

The first term in the denominator in Equation (S6) relates to the inductance of the planar spiral inductor, and the second term in the denominator attributes to the additional inductance due to the presence of the ferrite film [2,3].

If the thickness and edge length of ferrite film are t and a, and the initial distance between the ferrite film and planar spiral inductor is d_0, Equation (S6) can further be derived to the following formula [2]:

$$L_0 = \frac{N^2 \mu_0}{\frac{1}{t \mu_r} + \frac{d_0}{a^2} + C} \tag{S7}$$

.

C is the magnetic path in the air beyond the gap of the coil and ferrite film, which could be considered as a constant in the whole experiments [2].

Under the external load, the top membrane of the sensor deforms elastically. If the distance between the ferrite film and the planar spiral inductor decreases to d' under the external load, according to Equation (S7), the variation of inductance can be expressed as:

$$\Delta L = N^2 \mu_0 \left(\frac{1}{\frac{d'}{a^2} + \frac{1}{t\mu_r} + C} - \frac{1}{\frac{d_0}{a^2} + \frac{1}{t\mu_r} + C} \right) = L_0 \left[\frac{d_0 - d'}{d' + a^2 \left(\frac{1}{t\mu_r} + C \right)} \right] \tag{S8}$$

In the denominator, d' is much smaller than the second term, so it can be ignored. $d' - d_0$ can be substituted with Δd, and the relative inductance changes can be expressed as:

$$\Delta L / L_0 = \frac{d_0 - d'}{d' + a^2 \left(\frac{1}{t\mu_r} + C \right)} \approx \frac{\Delta d}{a^2 \left(\frac{1}{t\mu_r} + C \right)} \tag{S9}$$

Mechanical Deformation

Small deflections of the sensing membranes can be mathematically predicted according to the classic thin-plate theory. The relationship between the membrane deflection (Δd) and external force (ΔF) can be expressed as [4]:

$$\Delta d = \frac{(1-\nu^2) a^2}{5ET^3} \Delta F \tag{S10}$$

where v, E and T are the Poisson's ratio, Young's modulus and thickness of the sensing membrane, respectively. According to the relationship between force and pressure ($\Delta F = \Delta P \times a^2$), we get the following formula based on Equation (S10):

$$\Delta d = \frac{(1-\nu^2) a^4}{5ET^3} \Delta P \tag{S11}$$

Mechanical-to-Inductive Sensitivity

Combining Equations (S9) and (S11), the relative inductance changes ($\Delta L/L_0$) and the pressure (ΔP) have the following relationship:

$$\frac{\Delta L / L_0}{\Delta P} = \frac{a^2 (1-\nu^2)}{5ET^3 \left(\frac{1}{t\mu_r + C} \right)} \tag{S12}$$

Reference

1. Zhou, Z.; Yuan, H.J. *Principles and Applications of Sensors*, 1st ed.; Tsinghua University Press, Block A of Tsinghua University Research Building, Beijing, China, 2011, pp. 72.
2. Saidani, M.; Gijs, M.A.M.; Cubic Millimeter Power Inductor Fabricated in Batch-Type Wafer Technology. *J. Microelectromech. S.* **2003**, *12*, 172-178.
3. Su, Y.P.; Liu, X.; Hui, S.Y. Extended Theory on the Inductance Calculation of Planar Spiral Windings Including the Effect of Double-layer Electromagnetic Shield. *IEEE. T. Power. Electr.* **2008**, *23*, 2052-2061.
4. Nie, B.Q.; Xing, S.Y.; Brandtb, J.D.; Pan, T.R. Droplet-Based Interfacial Capacitive Sensing. *Lab. Chip.* **2012**, *12*, 1110-1118.
